# Quantitative Trait Loci Mapping for Powdery Mildew Resistance in Wheat Genetic Population

**DOI:** 10.3390/genes15111438

**Published:** 2024-11-06

**Authors:** Zhiyong Zhao, Yuliang Qiu, Menglin Cao, Hongyuan Bi, Guan Si, Xianghai Meng

**Affiliations:** 1 Institute of Cotton Research, Shanxi Agricultural University, Yuncheng 044000, China; 18903590243@126.com (Z.Z.); cmlin34@163.com (M.C.); 18135965766@163.com (H.B.); 18847161465@163.com (G.S.); 2 Dryland Farming Institute, Hebei Academy of Agriculture and Forestry Sciences, Hengshui 053000, China

**Keywords:** wheat, DH population, powdery mildew resistance, QTL mapping

## Abstract

Powdery mildew is a prevalent wheat disease that affects yield and quality. The characterization and fine mapping of genes associated with powdery mildew resistance can benefit marker-assisted breeding. In this study, quantitative trait loci (QTL) associated with powdery mildew were mapped using a high-density 35K DArT genetic linkage map developed from a population of double haploid lines (DHs) created by crossing “Jinmai 33 (a highly resistance line) with Yannong 19 (a highly susceptible line)”. Three stable QTLs for powdery mildew were identified on chromosomes 1B, 2B, and 6A combined with the composite interval graphing method and multiple interval mapping, explaining phenotypic variations (PVE) that range from 4.98% to 13.25%. Notably, *Qpm.sxn-1B* and *Qpm.sxn-2B* were identified across three environments, with the PVE ranging from 9.37% to 13.25% and from 4.98% to 5.23%, respectively. The synergistic effects of these QTLs were contributed by the parental line “Jinmai 33”. *Qpm.sxn-1B* was the major stable QTL, and *Qpm.sxn-2B* was close to *Pm51*. Furthermore, *Qpm.sxn-6A* was identified in two environments, accounting for PVE values of 7.13% and 7.65%, respectively, with the resistance effects originating from the male parent. Remarkably, this locus has not been reported previously, indicating that *Qpm.sxn-6A* represents a newly dis-covered QTL governing powdery mildew genes. Conclusions Five molecular markers available for mark-er-assisted selection were selected for tracking *Qpm.sxn-1B* and *Qpm.sxn-2B* in the program. The identification of this novel newly discovered QTL and markers reported in this study will be useful for marker-assisted selection of powdery mildew resistance.

## 1. Introduction

Wheat (*Triticum aestivum* L.) is a vital crop in China, ranking among the top three traditional food crops. It covers an extensive area of 23.38 million hectares and yielded approximately 130 million tons in 2020, and it is also ranked as one of the top three crops in China [1]. Given its significance, wheat production directly influences food security and the living standard of the Chinese population. However, its productivity is consistently threatened by myriad biotic and abiotic factors. Specifically, the prevalence of wheat powdery mildew (*Blumeria graminis* f. sp. *tritici*, Bgt) has gradually developed from a minor disease that occurs in spot patches to a major disease in China’s primary wheat-producing regions since the 1970s [1]. This development has posed a serious threat to wheat production. According to the National Agricultural Technology Extension Service Center, the occurrence area of wheat powdery mildew (*Pm*) in 2019 was estimated to be approximately 1 million hectares, with severe impacts being observed in the Huanghuai winter wheat region and certain areas along the middle and lower reaches of the Yangtze River [1]. The *Pm* not only decreases wheat yield, but also influences wheat processing and baking quality [2,3]. While chemical spraying and the use of resistant varieties are the primary control measures, prolonged fungicide application can lead to the development of pathogen resistance to fungicides. The prevalence of resistant strains had reached 97% since as early as 2013 [4]. Furthermore, chemical spraying also contributes to environmental pollution. Consequently, more environmentally friendly, economical, and sustainable approaches are needed to control wheat *Pm*. Breeding resistant wheat cultivars represents a more effective strategy to control *Pm* than the use of fungicides.

The identification and utilization of quantitative trait loci (QTL) associated with *Pm* resistance has the potential to accelerate the breeding of wheat varieties that exhibit resistance to this disease, thus enhancing the efficiency of a disease resistance breeding program. To date, more than 100 *Pm* resistance alleles have been identified and formally designated, including *Pm1*–*Pm68* [5,6,7], which distributed across all chromosome groups of wheat. Most of these genes are race-specific, which makes it easy to lose resistance with large-scale deployment in production due to the evolution of the pathogen. It has been reported that *Pm2*, *Pm3a*, *Pm3b*, *Pm3f*, *Pm4a*, *Pm6*, *Pm8*, and *Pm17* have been overcome in part or all of the USA, while *Pm1a*, *Pm3a*, and *Pm8* were defeated in Australia, China, and Egypt [8,9]. Moreover, most *Pm* genes exhibit resistance throughout the entire growth period, with only a few demonstrating resistance exclusively during the adult stage [10]. Therefore, it is necessary to continuously search for new *Pm* genes/loci to reply to the constantly evolved Bgt isolates.

Molecular markers are extensively utilized in the testing of *Pm* genes due to their simplicity and cost-effectiveness [11]. For example, *Pm12* in *Spertosaurus goatgrass* was identified based on 16 restriction fragment length polymorphism (RFLP) probes [12]. A sequence-characterized amplified region (SCAR) marker for the *Pm21* gene [13], which is currently one of the most effective resistance genes in China, has been used in wheat resistance breeding [14,15]. Fu et al. [16] utilized sequence-tagged site (STS) marker and cleaved amplified polymorphic sequence (CAPS) marker to precisely locate *Pm48* on the chromosome 5DS arm segment of wheat between the positions 0.63 and 0.67 Mb. Furthermore, Chen et al. [17] and Zhang et al. [18] applied bulked segregant analysis (BSA) to map the highly resistant *Pm* genes *PmCH1357* and *Pm64* to the chromosome 5DS and 2DL regions, respectively. The *QPm.caas-3BS* was mapped on chromosome 3BS in a Zhou8425B/Chinese Spring population with SNP marker, and three gene-specific kompetitive allele-specific PCR (KASP) markers were developed [19]. Numerous studies have demonstrated that the disease resistance conferred by a single QTL is often overcome by the evolution of *Pm* races. Therefore, the identification of novel sources of resistance and loci association with *Pm* and their polymerization in the disease resistance breeding program represents an effective strategy for the development of new wheat varieties with durable high resistance.

The *Pm* genes currently reported are derived from common wheat or its relatives, and nearly half of the reported *Pm* genes are derived from common wheat, including *Pm52*, *Pm59*, and *Pm65* [6], which could be directly applied in breeding practices through cross or backcross. Therefore, mining and utilizing novel genes/alleles from common wheat is more attractive to wheat resistance breeding. In the present study, the doubled haploid (DH) population derived from the highly resistant *Pm* cultivar “Jinmai 33” and the highly susceptible cultivar “Yannong 19” were utilized as experimental materials. A high-density genetic linkage map was constructed using the 35K DArT chip (Canberra, Australia; https://www.diversityarrays.com, accessed on 2 December 2018), and a QTL analysis of *Pm* at the adult stage was conducted. The aim was to provide a theoretical foundation for the identification and characterization of wheat *Pm* resistance genes and facilitate molecular marker-assisted (MAS) breeding.

## 2. Materials and Methods

### 2.1. Materials

The genetic population consisted of 184 DH lines derived from the wheat variety “Jinmai 33”, which exhibits high resistance to powdery mildew, and the highly susceptible powdery mildew variety “Yannong 19”. The varieties UIka/8 * Cc [20], KM2939 [21], and Birdwheat [22] were used as resistant checks, while the variety “Mingxian 169” [23] was utilized as the susceptible control line. All materials mentioned are stored in our group’s collection.

The DH population, two parents, and the resistant and susceptible control lines were rated for *Pm* response in the field at the experimental station of Liucun, Linfen, in Shanxi Province, China, during the 2015–2016 (E1) and 2016–2017 (E2) cropping seasons and at Yuncheng Cotton Institute Experimental Base in Shanxi Province during the 2016–2017 planting season (E3). All field trials were conducted in randomized complete block design (RCBD) with three replications. Each plot consisted of two 1.5 m rows spaced 0.3 m apart with 25 seeds per row. Planting was performed in early October of each year, with harvest taking place in mid-June of the subsequent year. Standard cultivation practices were employed throughout the study.

### 2.2. Identification of Pm Resistance

The resistance of DHs to powdery mildew were identified at experimental bases of Shanxi Normal University (Linfen, China) and the Cotton Research Institute of Shanxi Agricultural University (Yuncheng, China) in the 2015–2016 and 2016–2017 periods. The natural disease occurrence in the field was combined with artificial inoculation using the E09 physiological race. This inoculation took place after jointing, with three plants being inoculated for each strain. At the grain filling stage (14 days after flowering), the number of disease-resistant and susceptible plants were determined, while the infected control plant was fully diseased.

The resistance identification was conducted following the *Pm* resistance grading standard at the adult stage developed by Wang et al. [24]. Disease resistance was assessed based on leaf disease severity and reactive type using a five-point grading system. This scale is defined as follows: after heading, the whole plant is classified as disease-free, that is, the plant is resistant to disease or immunity, and this is classified as grade 0; the disease extends to the inverted four-leaf plant, that is, the plant is highly resistant, and this is classified as grade 1; the disease is extended to the inverted clover, that is, the plant has medium resistance, and this is classified as grade 2; the disease is extended to the inverted second leaf, i.e., the plant is medium-sensitive, and this is classified as grade 3; the flag leaf disease, i.e., plant high susceptibility, is classified as grade 4; and grade 5 indicates that the disease has reached the panicle. The survey was conducted twice, and the assessment was conducted based on the most severe disease observed.

### 2.3. Microscopic Observation of Pm Spore Hyphae

The leaves were collected at seven days after inoculation and immersed in a decolorizing solution consisting of 1.5 g·L^−1^ trichloroacetic acid alcohol solution (trichloromethane at a ratio of 4:1) for a duration of 48 h. Subsequently, the leaves were rinsed three times with double distilled water. To stain the leaves, a 1 g·L^−1^ cyanide solution was applied for a period of 10 s, followed by a quick rinse with double distilled water. Finally, the stained leaves were carefully positioned on a glass slide containing glycerol and covered with a cover slip. The observation and photography of the stained leaves were carried out using an Olympus CX-43 microscope.

### 2.4. Genotyping and Linkage Map Construction

DNA was extracted from all DH lines and parents using the CTAB method. And they were genotyped using a 35K DArT chip (Canberra, Australia; https://www.diversityarrays.com, accessed on 2 December 2018). Non-polymorphic markers in the molecular marker data were eliminated based on the genotypes of “Jinmai 33” and “Yannong 19”. All genotyped markers were filtered by excluding those either monomorphic or with high frequencies of missing values (>25%), or those with significant segregation distortion (χ^2^ test, *p* ≤ 0.05). The genetic map was constructed using QTL IciMapping V4.1 [25] and JoinMap 4.0 [26]. Markers were binned if the correlation coefficient between them was “one” using the BIN function in QTL IciMapping V4.1 according to the method reported by Winfield et al. [27]. The MSTMAP [28] was used to calculate the genetic distance between adjacent markers. Finally, MapChart 2.32 [29] was employed to generate genetic linkage maps.

### 2.5. QTL Analysis

The QTL was analyzed using Windows QTL Cartographer v2.5 combined with the Composite Interval Graphing Method (CIM) and Multiple Interval Mapping (MIM) [30]. Empirical threshold logarithm of odds (LOD) scores were calculated with 1000 permutations at *p* ≤ 0.05 [31], and the minimal LOD score to accept the presence of a QTL was set at 2.50. A QTL with a mean phenotypic variation explained (PVE) > 10% was defined as a major QTL, and one showing significance in at least two environments was defined as a stable QTL. The nomenclature for QTL follows the format Q+trait+location-chromosome name. The letter Q represents QTL, while the numbers 1 and 2 are appended to chromosome names to differentiate between QTL located on the same chromosome.

### 2.6. Marker Development and Evaluation of Markers for MAS

Resequencing was performed on Jinmai 33 and Yannong 19, and the insertion and deletion (Indel) variation existing between parents were analyzed based on the target interval of stable QTL. Polymorphic sequences containing InDel (>3bp) were converted to PCR-based markers using PrimerServer (http://wheatomics.sdau.edu.cn/PrimerServer/, accessed on 26 October 2024). According to the resequencing data of parents, the SNPs adjacent to the QTL LOD peak were preferentially picked out to develop KASP. And allele-specific and common primers for each KASP markers were designed using Primer3 software (http://biotools.umassmed.edu/bioapps/primer3_www.cgi, accessed on 26 October 2024). Genotype and phenotype identification were performed in DH offspring using those designed markers to verify the effectiveness of the markers.

The 12 powdery mildew-susceptible wheat cultivars/lines from different major wheat-producing regions and two parents, including Yannong 19, Jinmai 33, Jinmai 47, Yunhan 618, Jinmai 919, Yannong 187, Shannong 1538, Zhoumai 27, Zhongyu 1311, Yan 1212, Jimai 229, and Jimai 22 were tested by using the developed Indel and KASP markers. Additionally, we randomly selected lines from three validation populations, namely F_2_ (Jinmai 33/Jinmai 47) (50 lines), F_2_ (Jinmai 33/Yunhan 618) (50 lines), and F_3_ (Jinmai 33/Jinmai 919) (30 lines), to detect the utility marks, and the levels of response to *Pm* were observed for the corresponding line based on the marker’s polymorphism.

## 3. Results

### 3.1. Pm Resistance in DH Population

**Figure 1:**
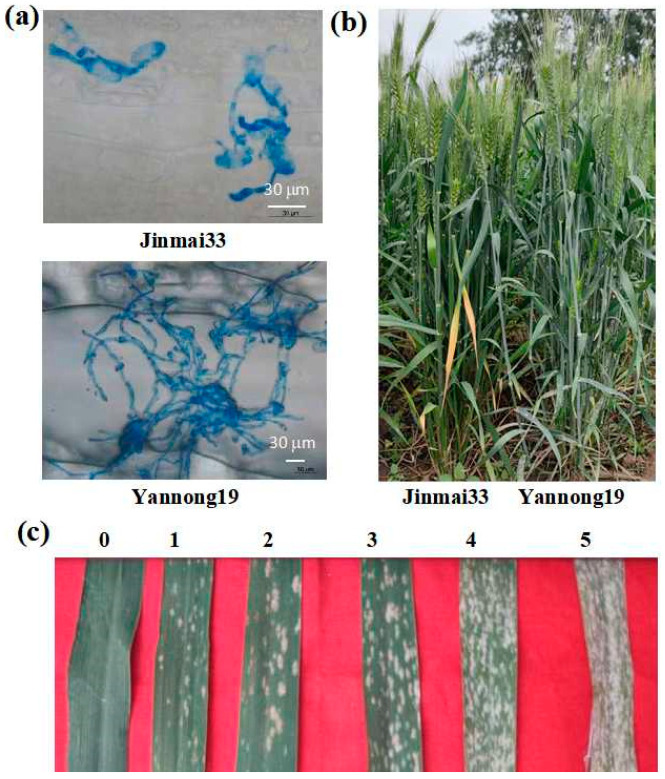
Disease resistance of parents and part of DH lines. (**a**) Microscopic observation of powdery mildew spore hyphae for Jinmai 33 and Yannong 19. (**b**) Disease susceptibility of adult plants between Jinmai 33 and Yannong 19. (**c**) Different grades of disease susceptibility of part adult plants in DH population.

The growth status and adult strain performance of the parental *Pm* spores can be observed (Figure 1). The results indicate that seven days after inoculation, the spores of *Pm* in the leaves of the resistant line “Jinmai 33” were found to be in the state of attached spore tubes with the absence of secondary hyphae. On the other hand, the spores of *Pm* in the leaves of “Yannong 19” exhibited the presence of secondary hyphae (Figure 1a). When inoculated with isolate E09 in the field trial, Jinmai 33 was highly resistant with IT 0, whereas Yannong 19 was highly susceptible, with the flag leaves being infested (Figure 1b). And the lines of the DH population from Jinmai 33 × Yannong 19 showed different levels of disease resistance (IT 0–5) (Figure 1c).

A positive and highly significant correlation coefficient of DH population resistance was observed across the three environments. Pearson’s correlation coefficients (r) among all three environments ranged from 0.55 to 0.58 (*p* < 0.01) (Table S1). The ANOVA indicated significant phenotypic variation among genotypes (G) and genotype by environments (G × E) (*p* < 0.001), and no significant variation was observed among environments (E) (Table S2). This indicated that both the genotype and G × E interaction had effects on powdery mildew resistance. The proportion of resistance to *Pm* in the DH population in different environments was further analyzed (Table 1). It was discovered that in the E1 environment, there were 157 lines classified as resistant (grades 0–2), while 27 lines were classified as disease-resistant (grades 3–5). In the E2 environment, 162 resistant lines and 22 susceptible lines were used. Similarly, in the E3 environment, there were 158 lines classified as resistant and 26 lines classified as susceptible. These findings suggest a susceptibility ratio close to 7:1, indicating that the disease resistance of this population may be controlled by three independent major resistance genes.

### 3.2. Construction of Genetic Map

Using a total of 36,420 DArT markers, we successfully detected polymorphisms within the DH population and its parental lines. From this analysis, we obtained 3867 non-redundant DArT markers. By leveraging these markers, we construct a comprehensive genetic map that covers all 21 chromosomes of wheat (Table 2). The resulting map spans a total length of 5920.93 cM, with an average marker density of 1.53 cM per marker.

A further investigation revealed substantial variations in marker density, the number of markers, and the lengths of chromosomes among the three sets of chromosomes (i.e., A/B/D) in wheat. The D genome had the lowest marker coverage, especially in chromosome 4D. The B chromosome group displayed the highest number of markers (1672), followed by the A chromosome group (1464), and the D chromosome group (731) (Table 2). These figures account for 43.24%, 37.86%, and 18.90% of the total markers, respectively, suggesting a pattern of B > A > D.

### 3.3. Genetic Loci of Pm Resistance

**Figure 2:**
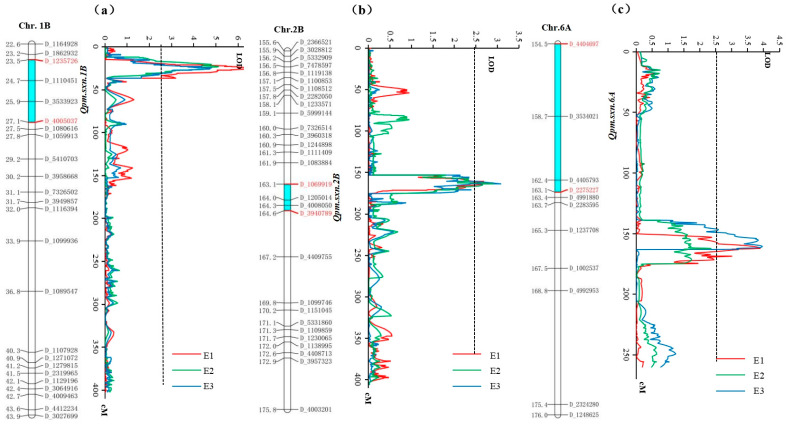
Genetic linkage map and graphical display of QTL for PM on chromosomes 1B (**a**), 2B (**b**) and 6A (**c**). Markers flanking the QTL are in red (Blue bars showed the positions of the QTL); Graphical display of QTL detected in separate trials are shown in different colors. The black dashed line represents the log-of-odds (LOD) score threshold of 2.50.

A total of six QTL for wheat powdery mildew resistance were detected on chromosomes 1B, 2B, 4B, 4D, 5A, and 6A with CIM. The LOD scores for these QTL ranged from 2.69 to 7.56, and the individual QTL explained 4.59–13.25% of the phenotypic variation in different environments (Table 3). Ten QTL for *Pm* were identified with MIM and PVE values in the range of 1.40–11.50% (Table S3). Notably, *Qpm.sxn-4B*, *Qpm.sxn-4D*, *Qpm.sxn-5A.1*, *Qpm.sxn-2B-2*, *Qpm.sxn-5D.2*, *Qpm.sxn-4D-1*, and *Qpm.sxn-4D-2* were exclusively detected in a single environment (Table 3 and Table S3). This observation indicates that these loci may be particularly susceptible to environmental influences. Alternatively, it raises the possibility that they may not represent statistically significant QTL but rather be artifacts of the analysis, potentially leading to false positives. Three QTL (*Qpm.sxn-1B*, *Qpm.sxn-2B*, and *Qpm.sxn-6A*) were found in two or more environments and identified both with CIM and MIM as stable QTL, distributed in chromosomes 1B, 2B, and 6A, respectively (Figure 2), which was also consistent with the results of the χ^2^ detection of *Pm* resistance. Except these stable QTL, *Qpm.sxn-3B.2* and *Qpm.sxn-4B* were identified both in E1 and E2 using MIM, with the PVE in the ranges of 3.40–9.90% and 3.90–6.20%, respectively (Table S3). Combining two QTL identification methods (CIM and MIM), *Qpm.sxn-1B*, *Qpm.sxn-2B*, and *Qpm.sxn-6A* were stable QTL for *Pm* in the DH population.

Taking the QTL results with CIM as an example, *Qpm.sxn-1B* was repeatedly detected in all three environments, which was located in the marker interval of *D_4005037*–*D_1235726* with LOD values of 6.32, 5.03, and 7.56, and it can explain 11.56%, 9.37%, and 13.25% of the phenotypic variance, respectively. They were identified as stable QTL with a negative additive allelic effect, indicating that the resistant allele came from the maternal parent “Jinmai 33” (Table 3). The PVE values of *Qpm.sxn-2B* were 5.18%, 5.23%, and 4.98%, in E1, E2, and E3, respectively. And the marker interval was *D_1069919-D_3940789*. The additive effect of *Qpm.sxn-2B* was contributed by the female parent (Table 3). The *Qpm.sxn-6A* was identified in E1 and E2, located in the marker interval of *D_4404697-D_2275227*, with PVE values of 7.13% and 7.65%, respectively, and the additive allelic effect from Yannong 19, a susceptible material (Table 3 and Figure 2). It may be a recessive control gene and need further verification. There is no report of a *Pm* resistance locus near *Qpm.sxn-6A* on chromosome 6A. Therefore, *Qpm.sxn-6A* potentially represents a novel, stable-effect QTL as it has not yet been found to co-locate with any of the QTL reported in previous studies.

Additionally, significant epistatic interactions were identified for *Pm* (Table S4). Interactions were observed in E1 and E2, and no significant interactions were detected in the E3. The largest epistatic interaction was observed for *Pm* between *Qpm.sxn-1B* and *Qpm.sxn-2B-1*, with the estimated additive-by-additive interaction effect of 2.40. but there was no epistatic effect observed for the three stable QTL regions of *Qpm.sxn-1B*, *Qpm.sxn-2B*, and *Qpm.sxn-6A* (Table S4).

### 3.4. Molecular Markers for MAS

**Figure 3:**
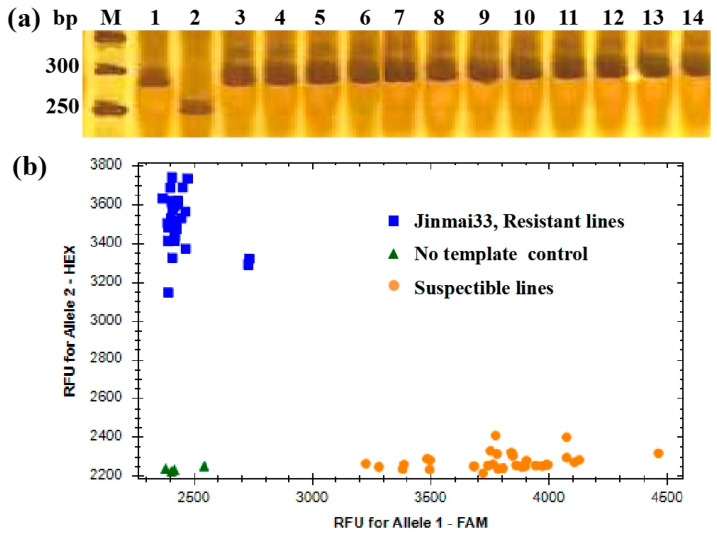
Amplification patterns of Qpm.sxn-1B-linked marker *Pm1B201* (**a**) in genotyping Yannong 19, Jinmai 33, and 12 other wheat cultivars susceptible to *Pm* and *KASP1B01* (**b**) in validation population. 1, Yannong 19; 2, Jinmai 33; 3, Jinmai 47; 4, Yunhan 618; 5, Jinmai 919; 6, Yannong 187; 7, Shannong 1538; 8, Zhoumai 27; 9, Zhongyu 1311; 10, Yan 1212; 11, Jimai 229; 12, Jimai 22.

Through the identification of the design markers within the DH population, four Indel markers (*Pm1B201*, *Pm1B209*, *Pm2B209*, and *Pm2B216*) and two KASP markers (*KASP1B01* and *KASP2B06*) associated with *Qpm.sxn-1B* and *Qpm.sxn-2B* were selected (Table S5). To better use two stable QTL (*Qpm.sxn-1B* and *Qpm.sxn-2B)* in MAS, six markers that are closely linked to *Qpm.sxn-1B* and *Qpm.sxn-2B* were evaluated for their effectiveness in Jinmai 33, Yannong 19, and other 12 susceptible wheat cultivars/lines for MAS. With the exception of *Pm2B204*, the remaining markers successfully amplified polymorphic bands between Jinmai 33 and the other susceptible varieties (Figure 3a). These findings indicate that these five markers can be used singly or in combination in MAS for tracking *Pm* when transferred into those cultivars.

Among them, *Pm1B201* and *KASP1B01* performed well in genotype differentiation and also distinguished between homozygous and heterozygous genotypes, which are suitable for MAS (Figure 3b). To further verify the validity of these two markers, 50, 50, and 30 lines from three breeding populations, namely F_2_ (Jinmai 33/Jinmai 47), F_2_ (Jinmai 33/Yunhan 618), and F_3_ (Jinmai 33/Jinmai 919), were identified, respectively. We found that only one line from F_2_ (Jinmai 33/Jinmai 47) and three lines from F_2_ (Jinmai 33/Yunhan 618) exhibited inconsistent genotypes and *Pm* resistance, and the four lines were possibly recombinants or powder hybrids. The results show that *Pm1B201* and *KASP1B01* are suitable for *Qpm.sxn-1B* in MAS, thereby different types of markers were provided to meet the needs of different detection.

### 3.5. The Additive Effect of the QTL on Pm Resistance

**Figure 4:**
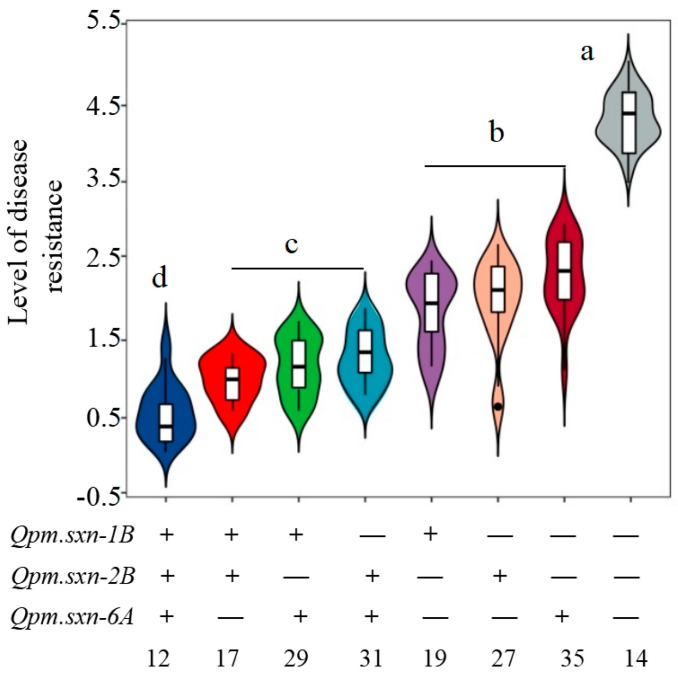
The disease levels for DHs with different QTL combinations in the Jinmai 33/Yannong 19 of 184 DH populations. The plus and minus represent lines with and without the positive alleles of the target QTL based on the flanking markers and the corresponding QTL. The numbers of lines carrying the corresponding number of favorable alleles are shown below the horizontal coordinate, and the letter above the bars indicate comparisons at the significant level of 0.05.

The Jinmai 33/Yannong 19 DHs were classified into four categories based on the number of QTL, namely those with none, one, two, and three QTL (Figure 4). Significant additive effects for *Pm* resistance were observed among these four categories, revealing that individuals within the population possessing the highest number of QTL exhibited enhanced stronger disease resistance (Figure 4). Plants that harbor three QTL favored alleles that displayed predominantly no disease symptoms. In contrast, lines with two and one QTL exhibited disease resistance scores ranging from 1 to 3, while lines with no QTL demonstrated a mean resistance level of 4.5, which is significantly higher than that of the other categories (Figure 4). These findings suggest that the presence of multiple QTL favored alleles associated with resistance, which contributed to an increased level of disease resistance.

## 4. Discussion

Powdery mildew, caused by Bgt, represents one of the most prevalent diseases affecting wheat with the potential to cause up to 40% grain loss or even worse during severe epidemics. The genes of wheat resistance to *Pm* mainly derived from three categories: common wheat, wild relatives of wheat, and wheat distal relatives. However, not all genes associated with disease resistance can be directly utilized in wheat breeding programs, as some may exhibit adverse pleiotropic effects that can impact their effectiveness. Furthermore, factors such as linkage redundancy and competition lag also play significant roles in limiting the effective utilization of disease resistance genes [32]. Generally, the genes derived from the wild relatives of wheat cannot be directly applied in wheat production due to the poor agronomic traits or undesirable linkage drag. For instance, while the *Pm16* gene provides a high level of resistance, it is associated with a reduced yield, and it is difficult to use it in production [32]. At present, the rye 1RS chromosome harbors the powdery mildew resistance gene *Pm8* in addition to the tightly linked rust resistance genes *Yr9*, *Lr26*, and *Sr31*. This genetic composition makes it widely used worldwide due to its adaptability and the yield enhancement it provides [33]. However, the *Sec-1* gene located on the 1RS chromosome can reduce dough elasticity and the processing quality of bread, thereby limiting the use of materials containing the 1RS translocation line [34]. Furthermore, some genes derived from distant wheat species (tertiary gene sources) require significant time and effort to improve in order to achieve the yield levels observed in commercially promoted varieties. In contrast, *Pm* genes derived from common wheat can be directly applied to breeding practices, such as *Pm52*, *Pm59*, and *Pm65* [6]. This approach will establish a foundation for the utilization of resistance genes, molecular marker-assisted breeding, fine mapping, rapid breeding [35,36], and gene editing [37].

In this study, the DH population derived from “Jinmai 33” × “Yannong 19” was utilized as genetic material, facilitating the application of QTL in breeding programs. This study revealed that the *Pm* resistance in this population were controlled by three pairs of main genes, corresponding to chromosomes 1B, 2B, and 6A. The PVE of *Qpm.sxn-1B* and *Qpm.sxn-2B* in the three environments ranged from 9.37% to 13.25% and from 4.98% to 5.23%, respectively. The resistance of these two QTL was found to have originated from “Jinmai 33”. Jinmai 33 was derived from a cross of Pingyang 79391 × Pingyang 79262, and Pingyang 79391 was derived from Naixue//036/3/76-1295; Pingyang 79262 is a hybrid of Weidong 7 and Xiangyang 4 with MaZhamai pedigree descendants [38]. These strains are based on “PingyaoXiaobaimai” and “Mazhamai” and then hybridized with excellent foreign varieties multiple times [39,40]. Therefore, the wide range of parental sources and the rich genetic diversity contribute to the presence of multiple *Pm* resistance loci in “Jinmai 33”. A further analysis revealed that the location of *Qpm.sxn-2B* (744.16 Mb – 765.48 Mb) is adjacent to *Pm51* (709.82 Mb–739.39 Mb), suggesting a possible relationship that needs verification through SSR or EST-PCR markers [41].

QTL *Qpm.sxn-6A* was detected in two environments, with the resistance effect originating from “Yannong 19”(Table 3), a material classified as susceptible [42]. It is speculated that this locus may be a recessive control gene [43,44,45]. This is consistent with the findings of Li et al. [46], who identified a single recessive gene, *PmQ*, in Qingxinmai, which confers seedling resistance to powdery mildew. Moreover, the *Qpm.sxn-6A* has not been identified in any of the previously reported powdery mildew resistance genes, which means it may be a new *Pm*-resistant locus. Additionally, the development of molecular markers associated with these loci will promote the breeding of disease-resistant cultivars. In this study, we evaluated the availability of markers linked with QTL *Qpm.sxn-1B* and *Qpm.sxn-2B* across 12 susceptible commercial cultivars and three breeding populations. These results indicate that five markers could be used individually or in combination for MAS for tracking *Qpm.sxn-1B* and *Qpm.sxn-2B* in the background of those susceptible cultivars. However, no marker associated with *Qpm.sxn-6A* was screened. This may be because the localization interval of QTL is large, and the additive effect comes from the infected parent. Further research is needed to fine-map and identify the candidate gene for *Qpm.sxn-6A*.

## 5. Conclusions

A total of six and ten QTL related to *Pm* resistance were identified with CIM and MIM, respectively. Three stable QTL were identified on chromosome 1B,2B, and 6A both with two methods. Among them, *Qpm.sxn-1B* and *Qpm.sxn-2B* were consistently detected across all environments. The *Qpm.sxn-6A* was detected in both environments and showed resistance effects originating from the paternal parent “Yannong 19”, which may be a potentially novel locus for *Pm* resistance. Moreover, five molecular markers available for marker-assisted selection were identified for tracking *Qpm.sxn-1B* and *Qpm.sxn-2B* in breeding programs.

## Figures and Tables

**Table 1:** Genetic analysis/segregation ratios of powdery mildew resistance genes.

Parent/Population	Total Number of Plants	Disease Resistant	Disease Susceptibility	Expected Separation Ratio	χ^2^
Jinmai 33	20	20	0		
Yannong 19	20	0	20		
Jinmai 33 × Yannong 19 (E1)	184	157	27	7:1	0.80
Jinmai 33 × Yannong 19 (E2)	184	162	22	7:1	0.05
Jinmai 33 × Yannong 19 (E3)	184	158	26	7:1	0.45

**Table 2:** Marker density and chromosome distribution.

Chromosomes	Number of Loci	Length (cM)	Density (cM)/Marker
1A	160	228.02	1.43
2A	302	401.25	1.33
3A	206	355.42	1.73
4A	231	377.97	1.64
5A	70	104.91	1.50
6A	143	260.59	1.82
7A	352	454.54	1.29
1B	347	413.53	1.19
2B	328	406.86	1.24
3B	137	259.43	1.89
4B	80	170.39	2.13
5B	202	308.98	1.53
6B	219	364.51	1.66
7B	359	448.68	1.25
1D	118	211.22	1.79
2D	104	208.70	2.01
3D	122	224.07	1.84
4D	45	102.23	2.27
5D	104	261.79	2.52
6D	94	156.55	1.67
7D	144	201.29	1.40
A genome	1464	2182.70	1.49
B genome	1672	2372.38	1.42
D genome	731	1365.85	1.87
Total	3867	5920.93	1.53

**Table 3:** QTL for powdery mildew resistance in DH population with CIM.

QTL	Env.	Chr.	Flanking Markers	Genetic Distance (cM)	LOD Value	Phenotypic Variance (PVE) (%)	Additive Effect
*Qpm.sxn-1B*	E1	1B	*D_4005037-D_1235726*	23.48–27.14	6.32	11.56	−0.40
	E2	1B	*D_4005037-D_1235726*	23.48–27.14	5.03	9.37	−0.37
	E3	1B	*D_4005037-D_1235726*	23.48–27.14	7.56	13.25	−0.56
*Qpm.sxn-2B*	E1	2B	*D_1069919-D_3940789*	163.06–164.60	2.74	5.18	−0.27
	E2	2B	*D_1069919-D_3940789*	163.06–164.60	3.16	5.23	−0.29
	E3	2B	*D_1069919-D_3940789*	163.06–164.60	3.02	4.98	−0.21
*Qpm.sxn-4B*	E2	4B	*D_1395268-D_1234524*	39.83–41.39	2.69	4.59	0.27
*Qpm.sxn-4D*	E1	4D	*D_1121275-D_4991578*	106.35–117.92	4.17	7.89	0.33
*Qpm.sxn-5A.1*	E2	5A	*D_1320425-D_1013062*	123.58–114.62	4.47	7.92	0.34
*Qpm.sxn-6A*	E1	6A	*D_4404697-D_2275227*	154.54–163.06	3.68	7.13	0.32
	E2	6A	*D_4404697-D_2275227*	154.54–163.06	3.92	7.65	0.35

## Data Availability

The original contributions presented in this study are included in the article; further inquiries can be directed to the corresponding authors.

## Supplementary Materials

The following supporting information can be downloaded at https://www.mdpi.com/article/10.3390/genes15111438/s1, Table S1: Correlation of disease resistance among DH population across three environments; Table S2: Analyses of variance (ANOVA) for powdery mildew resistance in DH population across multiple environments; Table S3: QTL for powdery mildew resistance with multiple interval mapping; Table S4: Epistatic QTL analysis for powdery mildew resistance with multiple interval mapping; Table S5: The markers linked to QTL *Qpm.sxn-1B* and *Qpm.sxn-2B*.
